# Semifield Evaluation of Improved Passive Outdoor Host Seeking Device (POHD) for Outdoor Control of *Anopheles arabiensis* Mosquitoes

**DOI:** 10.1155/2020/8938309

**Published:** 2020-05-26

**Authors:** Stella T. Kessy, Bruno A. Nyundo, Ladslaus L. Mnyone, Issa N. Lyimo

**Affiliations:** ^1^Department of Environmental Health and Ecological Sciences, Ifakara Health Institute, Off Mlabani Passage, P.O. Box 53, Ifakara, Morogoro, Tanzania; ^2^Department of Zoology and Wildlife Conservation, College of Natural and Applied Sciences, University of Dar es Salaam, P.O. Box 35064, Dar es Salaam, Tanzania; ^3^Pest Management Centre, Sokoine University of Agriculture, P.O. Box 3110, Morogoro, Tanzania; ^4^School of Public Health, Faculty of Health Sciences, University of the Witwatersrand, Johannesburg, South Africa

## Abstract

Despite the considerable progress made so far, the effectiveness and mass application of odour-baited outdoor mosquito control devices in pipelines is limited by several factors. These include the design and size of the devices, optimal placement of attractive blends, and nature of materials into which the blends are impregnated. The primary aim of this study was to manipulate these factors to improve the attractiveness of our recently developed passive outdoor host seeking device (POHD) to outdoor biting *Anopheles arabiensis*. Specifically, the study aimed to determine optimal placement of odour blends and killing bioactives in POHD for maximum attraction and killing of *An. arabiensis* and to assess the effects of blend types, formulation, and residual activity on attractiveness of the POHD to *An. arabiensis*. The POHDs baited with attractive blends, carbon dioxide (CO_2_), and bendiocarb-treated electrostatic netting were placed either towards the top or bottom openings, and other modifications were exposed to *An. arabiensis* under the semifield system at Ifakara Health Institute (IHI). Each night, a total of 100 starved female, 3–7-day-old, semifield reared *An. arabiensis* mosquitoes were released, collected the next morning (alive or dead), counted, and recorded. Live mosquitoes were maintained in the semifield insectary and monitored for 24 hours mortality. Each treatment combination of the POHD was tested in three replicates. Overall, the results indicated that the proportion of mosquitoes attracted to and killed in the POHD varied with position of attractants and killing agent (bendiocarb). The POHD with bottom placed attractants and bendiocarb attracted and killed higher proportion of mosquitoes compared to the POHD with top placed attractants and bendiocarb. The highest mortalities were observed when the POHD was baited with a combination of attractive blends and CO_2_. Moreover, the residual activity of attractive blends applied inside POHD varied with type and formulation of attractive blend. The POHD packed with Mbita and Ifakara blend in microencapsulated pellets (granules) attracted higher proportion of mosquitoes than that baited with soaked nylon-strip formulation of either blends. Interestingly, POHD baited with Mbita blend in microencapsulated pellets (granules) formulation attracted and killed higher proportion of mosquitoes (>90%) than that baited with Ifakara blend even 9 months after application. Conclusively, the POHD remained effective for a relatively longer period of time when baited with bottom placed synthetic blends and CO_2_ combination, thus warranting further trials under real life situations.

## 1. Introduction

The current control of malaria vectors relies heavily on the use of long-lasting insecticidal nets (LLINs) and indoor residual spraying (IRS) [1, 2]. These vector control interventions alongside improved diagnosis and treatment with artemisinin-based combination therapy (ACTs) have significantly reduced malaria cases and deaths in many endemic countries [3–6]. However, the sustainability of LLINs and IRS is constrained by several factors, the most important of which include inability to target insecticide resistant and/or outdoor biting malaria vectors [3, 7, 8]. Since both of these interventions are based exclusively indoors, they miss outdoor and/or early biting vector species such as *Anopheles arabiensis* [6, 7, 9]. These vector species are increasingly dominating the malaria vector populations, thus maintaining residual malaria transmission in most of Africa [10, 11]. Therefore, if we need to safeguard the current malaria control gains and accelerate towards elimination, complementary outdoor-based control measures capable of targeting outdoor biting and/or insecticide resistant malaria vectors are desirable [6, 12, 13].

Several odour-baited outdoor-based control/surveillance devices have been developed and proof-tested under semifield and field settings [14–20]. These devices have demonstrated very promising results; however, most of them are expensive, bulk, and require power source and sophisticated skills to operate [18, 21, 22]. These hinder their large-scale trials and deployment particularly in marginalised and resource poor settings, which constitute the majority of endemic countries.

The odour-baited devices rely on several cues that mosquito vectors use to detect and locate their preferential hosts [23]. The most important cues include skin odours and carbon dioxide [24, 25]. These odours have been synthesised and constituted into attractive blends, for example, Ifakara blend [20], Mbita blend [26], and BG lures [26–28]. These blends have also been tested in combination with CO_2_ to enhance attractiveness and deployability in outdoor-based devices [14, 15, 19, 26, 29–31]. The attractiveness of blends and outdoor-based devices is influenced by several factors including design and size of the device, placement of blends inside the device, and nature of materials into which the blend is impregnated. The present study aimed to improve the attractiveness of passive outdoor host seeking device (POHD) we recently developed by manipulating some of the above factors. Specifically, we aimed to (1) identify optimal placement of attractive blends in a POHD for maximum attraction and killing of visiting malaria mosquitoes and (2) compare the residual activity of blends applied on nylon strips and granules on attracting malaria vectors to POHDs.

## 2. Materials and Methods

### 2.1. Study Site

**Figure 1:**
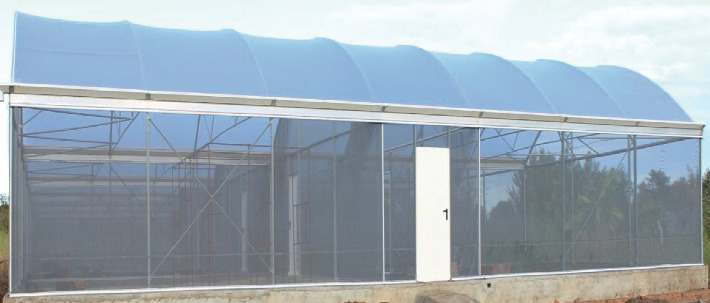
Picture of the semifield system (SFS) located at Ifakara Health Institute in Kilombero Valley, Southeastern Tanzania.

Experiments were conducted in the semifield system (SFS, Figure 1) at Kining'ina village, Kilombero valley (8.11417 S, 36.67864 E), Southeastern Tanzania, about 6 km from Ifakara town. The SFS is separated into several independent chambers (each 2.97 × 6.70 × 2.80 m), within which the experiments were replicated. Temperature inside the SFS over the period of the experiments ranged from 26 to 32°C.

### 2.2. Rearing of Experimental Mosquitoes

All experiments were conducted against unfed female *An. Arabiensis*, 3–7 days of age, reared inside the semifield system. The mosquito colony was originally established in 2008 from eggs of individuals collected from Sagamaganga village in Kilombero valley [32–34]. The malaria vectors population in this village is predominantly *An. arabiensis* (>95%) [35, 36]. Rearing of the mosquitoes was done per procedures described by Lyimo [34]. Larvae were reared in plastic basins (diameter 43 cm, depth 15 cm) and fed on TetraMin® that was finely ground baby fish food flakes (Tetra GmbH, Herrenteich 78, D-49324 Melle, Germany). Adults were reared in screened cages (45 × 45 × 45 cm) and provided ad libitum access to 10% glucose solution. Temperature ranged from 26 to 32°C. Adults of the parental stock were provided with human blood through arm feeding. The experimental female mosquitoes were never fed on blood (unfed).

### 2.3. Improved Passive Outdoor Host Seeking Device (POHD)

**Figure 2:**
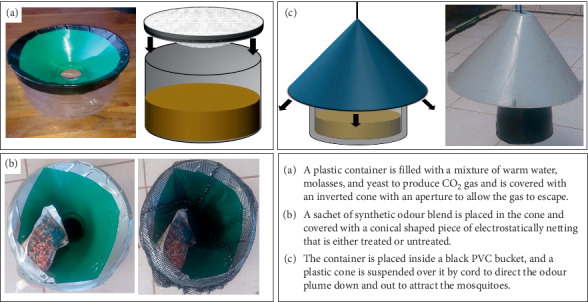
Improved POHD with top placement of attractants and bioactives. (a) Plastic jug filled with mixture of warm water, molasses, and yeast for production of CO_2_. (b) Sachet of synthetic odour placed on the cone, covered by bendiocarb-treated or untreated netting. (c) Plastic container placed inside PVC with a plastic cone suspended over it to allow odour plumes flowing downward.

**Figure 3:**
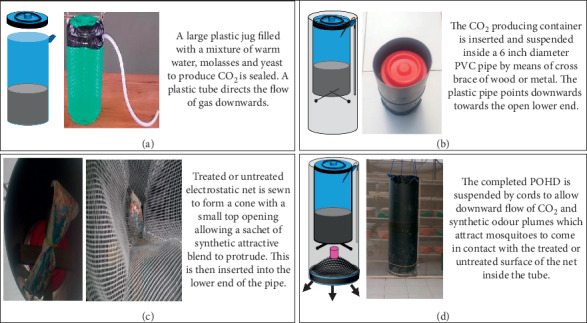
Improved POHD with bottom placement of attractants and bioactives. (a) Plastic jug filled with mixture of warm water, molasses, and yeast required for generation of CO_2_. (b) CO_2_ producing container inserted inside 6-inch diameter PVC pipe for transferring CO_2_ into the mouth of device. (c) Bendiocarb-treated or untreated netting with an opening showing synthetic attractive blend. (d) Completed POHD suspended by cords allowing downward flow of CO_2_ and synthetic odour plumes to attract and kill *Anopheles arabiensis*.

**Figure 4:**
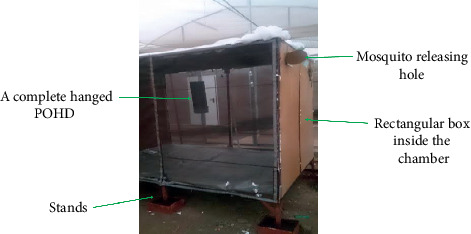
A bioassay box (187 × 212 × 115 cm) evaluating efficacy of POHD placed in the middle of a semifield chamber. The POHD is incorporated with different formulations of different synthetic attractive blends of different storage and usage period.

The POHD improved herein was designed and preliminarily evaluated in a previous study by Kessy et al. (unpublished). This POHD was improved using locally available polyvinyl chloride (PVC) pipe (0.16 × 0.47 m) and different placement and formats of the following components: (1) inner plastic jug of 2 L volume made by Cello Industries Tanzania Limited (0.05 × 0.09 m) for the mixture of molasses (Kilombero Sugar Company Limited, Kilombero, Tanzania) and Dry Instant Yeast (Pasha 450 Instant Yeast, Akmaya Group, Ruse, Bulgaria and Odessa, Ukraine) to generate carbon dioxide (CO_2_) required for these experiments (Figures 2(a) and 3(a)); (2) inner tube (0.01 m diameter and 0.4 m length) for release of CO_2_ to outside the device (Figure 3(a)); (3) inner bag/sachet/strips of synthetic attractive blends (Figures 2(b) and 3(c)); (4) inner conical shaped electrostatically charged netting to allow the flow of plume of odour and CO_2_ to outside the device (Figures 2(b) and 3(c)); and (5) outer PVC cover to protect inner components including the mosquito killing bioactives which were used as a proxy for mosquitoes visiting the device (Figures 2(c) and 3(c)).

Two different prototypes of POHD were constructed and experimented: top mosquito entry POHD (Figures 2(a) and 2(b)) and bottom mosquito entry POHD (Figure 3(a)). For the top mosquito entry POHD, attractive blends were placed towards the top opening of the device, while the bottom opening was tightly closed to ensure that the odour plumes and CO_2_ flow upwards to the top conical plastic cover. Thus, the plume of odours and CO_2_ hits the conical plastic cover and creates downwind flow that attracts mosquitoes to enter the device from the top (Figure 2(c)). For the bottom mosquito entry POHD, attractive blends and CO_2_ were placed towards the bottom opening while the top opening was tightly closed (Figure 3(b)). The tube of CO_2_ was placed such that CO_2_ was released directly onto the attractive blend. Because the top of the POHD is tightly closed, the lower compartment of the device becomes saturated with plume of odours and CO_2_ that easily flow downward and attract mosquitoes to enter via the bottom opening (Figure 3(d)). For both the top and down entry POHD, the bendiocarb-treated netting was placed on respective positions of the attractive blends.

### 2.4. Synthetic Blends and Bioactives inside POHD

Two synthetic blends were used in these experiments: Mbita blend (MB5) [37] and Ifakara blend (Ib) [38]. Mbita blend was originally developed and tested in Western Kenya, and it was composed of five different compounds (i.e., 2.5% aqueous ammonia, 85% L-lactic acid, 0.00025% tetradecanoic acid, 0.000001% methyl-1-butanol, and 0.000001% butylamine) as described by Mukabana et al. [37]. Ifakara blend was originally designed and tested at Ifakara Health Institute in Tanzania, and it was prepared using nine different compounds (i.e., 2.5% aqueous ammonia, 85% L-lactic acid, 0.01% tetradecanoic acid, 0.10% propionic acid, 1% butanoic acid, 0.01% pentanoic acid, 0.01% heptanoic acid, 0.01% octanoic acid, and 0.001% 3-methyl-1-butanoic acid) as described previously [38]. Between experiments, the blends were stored in the refrigerator (−4°C). Either of the blends was employed in the POHD in combination with CO_2_ in order to enhance attractiveness to mosquitoes. The CO_2_ used in these experiments was generated from a mixture of 1 L of warm water (37°C) [39, 40], yeast (8.75 g), and molasses (250 g) [41]. Such ratio of molasses and dry yeast in the mixture was derived based on evidence from previous studies which assessed effects of different quantities of carbohydrates (i.e., molasses, honey, and sugar) and dry yeast in a total volume of ≥0.1 L of warm water [39, 42–45], or ≥1 L of warm water [40, 41], on the release of optimum CO_2_ for at least an overnight attraction of mosquitoes. Powder formulation of bendiocarb (Ficam D) applied on electrostatically charged netting [46] was employed as a bioactive marker for killing mosquitoes visiting the POHD.

## 3. Experimental Procedures

### 3.1. Effect of the Placement of Attractants on Efficacy of POHD

We compared the attractiveness of POHDs with blends placed towards either the top or bottom opening of the PVC tube. The treatment combinations were as follows: (1) Mbita blend + CO_2_ without bendiocarb-treated net (Mb + CO_2_), (2) CO_2_ alone with bendiocarb-treated netting (CO_2_ + Be), (3) Mbita blend alone with bendiocarb-treated netting (Mb + Be), and (4) Mbita blend + CO_2_ with bendiocarb-treated netting (Mb + CO_2_ + Be). During each experimental night, POHD with either top or bottom placed blends was assembled and hung 25 cm off the ground, at the middle of the semifield system chamber. This height of 25 cm for hanging POHD from ground was selected because it is within the recommended range of heights (15–30 cm) for maximum mosquito catches in several odour-baited traps especially Mosquito Magnet X and Suna traps [16, 47]. During each experimental night, 100 female mosquitoes starved for 6 hours were released in groups of 25 mosquitoes at four different corners inside the SFS chamber. In the morning, live and dead mosquitoes inside the POHD and elsewhere within the SFS chamber were recovered, counted, and recorded. Live mosquitoes were maintained under 10% glucose solution within the semifield insectary and monitored for 24 hours mortality. The experimental SFS chamber was thoroughly cleaned with tap water, and any remaining mosquitoes were collected using a CDC Backpack aspirator with 12-volt battery (Model 1412, John W. Hock Company, USA) to prevent carryover effect. Each treatment combination in both the top and bottom placed blends was replicated three times. The different treatments were alternated daily over 12 consecutive nights adding up to a total of 1200 mosquitoes released over that period.

### 3.2. Assessing Residual Effect/Persistence of Blends

The improved POHD was used to evaluate the efficacy of Mbita (Mb) and Ifakara (Ib) blend, impregnated in different substrates (polymer pellets/granules formulation in sachets delivery format vs. liquid formulation in soaked nylon strips (26.5 × 1 cm) delivery format) over time after treatment “residual effect/persistence”. The residual effect/persistence was measured based on the number of mosquitoes attracted to, and killed at, the POHD. The residual activity/persistence of either blend in granules (polymer pellets) packed in sachets was assessed at three time intervals: one month, six months, and nine months after preparation. For blends soaked in nylon strips, the residual activity/persistence of either blend was assessed at two time intervals: one month and nine months after preparation. These blends were tested inside POHD in combination with CO_2_. The treatment combinations were as follows: (a) blend + CO_2_ + untreated netting, (b) bendiocarb-treated netting alone (Be), and (c) CO_2_ + bendiocarb-treated netting (CO_2_ + Be). These treatment combinations were retested at 1 mo, 6 mo, and 9 mo. The POHD was assembled and hung at the middle of the bioassay box (1.87 × 2.12 × 1.15 m, Figure 4), placed in the middle of SFS chamber (Figure 3). The bioassay box was erected on 4 stands that were kept in bowls with water to prevent ants. A total of 100 mosquitoes were released during each experimental night and left to forage for overnight from 7:00 pm to 6:00 am. In the morning, all mosquitoes found dead or alive inside the bioassay box and the POHD were recovered, counted, and recorded. All live mosquitoes were maintained inside the SFS insectary, provided with 10% glucose solution, and monitored for 24 hours' mortality. Each treatment combination was replicated three times.

### 3.3. Ethical Considerations

Ethical clearance was obtained from the Institutional Ethics Review Board (IRB) of Ifakara Health Institute (ref: IHI/IRB/No. 14-2013) and the Medical Research Coordinating Committee at the National Institute for Medical Research in Tanzania (ref: NIMR/HQ/R.8a/Vol. IX/1784). Also, the permission to publish this work was granted by the National Institute for Medical Research in Tanzania (NIMR/HQ/P.12 Vol XXVIII/77).

### 3.4. Statistical Analysis

Statistical analysis was conducted to confirm whether or not the efficacy of improved POHD depends on the following: (i) the optimal placement of synthetic attractive blends, CO_2_, and bioactives and (ii) the residual activity of different formulations of the blends (liquid soaked nylon strips and polymer pellets/granules). The efficacy of POHD was assessed based on the proportion of mosquitoes attracted and killed inside the POHD as the response variable. The response variable measured in these experiments was binomial (proportion of dead mosquitoes). Therefore, the relationship between this response variable and explanatory variables (treatments, blend types, and formulation types) was analysed using generalised linear mixed effect models with binomial errors (glmer) in the R statistical software package. The treatments, blend type, and formulation type in the device were taken as the main effect (fixed effects), and the replicates were taken as the random effect. A base model including only random effect of “replicate” was constructed. A sequential addition of the “main effects” and their interaction (treatment^∗^blend type, and treatment^∗^formulation type) was conducted to construct a maximal model (forward stepwise approach). A statistical significance of fixed effects and interaction term was generated and evaluated using likelihood ratio tests (LRTs). When the interaction terms were statistically significant, the main effect of formulation types for each synthetic attractive blend was analysed separately to generate estimates of the response variable. Then, full model was used to perform a two-way multiple comparison using Tukey post hoc tests (adjusting for multiple comparison) to establish statistical significant differences between treatments.

## 4. Results

### 4.1. Effect of the Placement of Attractants on Efficacy of POHD

**Figure 5:**
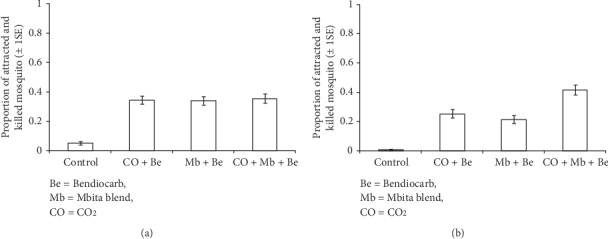
Estimated proportion (±1SE) of *An. arabiensis* mosquitoes that were killed after exposure to untreated or bendiocarb-treated passive host seeking device baited with attractants within the semifield system. (a) Top placement of attractants. (b) Bottom placement of attractants. Error bars represent plus/minus 1 standard error.

The proportion of mosquitoes attracted and killed by the POHD incorporated with different treatment combinations was significantly influenced by the position of blends (treatment^∗^position: *χ*_3_^2^ = 23.96, *P* < 0.001, Figures 5(a) and 5(b)). The efficacy of POHD baited with attractive blends placed towards the top opening varied significantly between treatments (*χ*_3_^2^ = 118.26, *P* < 0.001, Figure 5(a)). Multiple comparisons indicated that POHD without bendiocarb-treated netting killed significantly fewer mosquitoes than POHD with CO_2_ + Be (*z* = −7.68, *P* < 0.001), Mb + Be (*z* = 7.57, *P* < 0.001), and CO_2_ + Mb + Be (*z* = −7.82, *P* < 0.001). However, the proportion of dead mosquitoes was not significantly different between POHD with and without bendiocarb-treated netting (Figure 5(a)).

In contrast, the efficacy of POHD with attractive blends placed towards the bottom opening varied significantly with treatments (*χ*_3_^2^ = 161.88, *P* < 0.001, Figure 5(b)). The POHD without bendiocarb-treated netting had significantly fewer mosquitoes than POHD with bendiocarb-treated netting, CO_2_ + Be (*z* = −5.59, *P* < 0.001), Mb + Be (*z* = 4.71, *P* < 0.001), and CO_2_ + Mb + Be (*z* = −6.96, *P* < 0.001). Contrary to the top entry POHD, the bottom entry POHD with CO_2_ + Mb + Be attracted and killed higher proportion of mosquitoes than POHD with CO_2_ + Be (*z* = 4.07, *P* < 0.001) and Mb + Be (*z* = −6.18, *P* < 0.001, Figure 5(b)). However, the proportion of dead mosquitoes was similar between POHD with Mb + Be and CO_2_ + Be (*z* = −2.26, *P*=0.09, Figure 5(b)).

### 4.2. Residual Effect/Persistence of Attractive Blends

**Figure 6:**
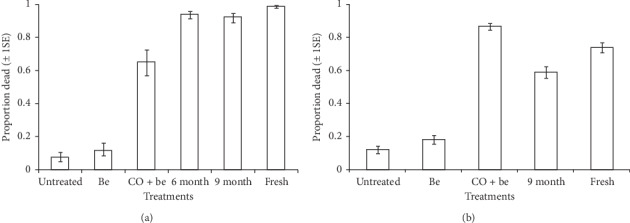
Estimated proportion (±1SE) of *An. arabiensis* mosquitoes that were attracted and killed after exposure to a passive host seeking device that was untreated or treated with bendiocarb and baited with different formats of Mbita blend, (a) microencapsulated polymer pellets/granules, (b) soaked nylon strips. Error bars represent plus/minus 1 standard error.

**Figure 7:**
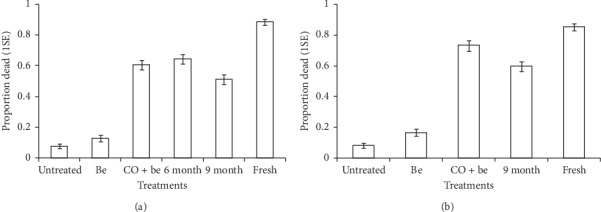
Estimated proportion (±1SE) of *An. arabiensis* mosquitoes that were attracted and killed after exposure to a passive host seeking device that was untreated or treated with bendiocarb and baited with different formats of Ifakara blend, (a) microencapsulated polymer pellets/granules, (b) soaked nylon strips. Error bars represent plus/minus 1 standard error.

The residual activity of synthetic blends was dependent on blend types for each application substrate (treatment^∗^substrate type: microencapsulated (pellets) granules, *χ*_6_^2^ = 220.55, *P* < 0.001; soaked nylon strips, *χ*_5_^2^ = 29.10, *P* < 0.001; Figures 6 and 7). Also, the residual activity of synthetic blends was significantly dependent on the type of application substrate for either of the tested blends (treatment^∗^blend type: Mbita, *χ*_5_^2^ = 16.49, *P* < 0.01; Ifakara, *χ*_5_^2^ = 16.49, *P* < 0.01; Figures 6 and 7).

In the POHD baited with Mbita blend in microencapsulated pellets (granules), the proportion of attracted mosquitoes varied significantly with treatments (*χ*_5_^2^ = 1154.8, *P* < 0.001, Figure 6(a)). The POHD with fresh granular formulation of Mbita blend attracted and killed higher proportion of mosquitoes compared to the POHD baited with six-month-old granular formulation (*z* = −14.72, *P* < 0.001), nine-month-old granular formulation (*z* = 3.53, *P* < 0.01), Be + CO_2_ (*z* = 6.92, *P* < 0.001), with Be (*z* = 11.74, *P* < 0.001), and without Be (*z* = −12.32, *P* < 0.001). Similar proportion of mosquitoes was attracted by POHDs baited with six-month-old and nine-month-old granular formulation (*z* = 0.84, *P* = 0.96). However, this proportion was significantly higher compared to that of POHD with CO_2_ + Be (*P* < 0.001),with Be (*P* < 0.001), and without Be (*P* < 0.001, Figure 6(a)).

In the POHD baited with Mbita blend in soaked nylon strips, the proportion of mosquitoes attracted to the device varied significantly with treatments (*χ*_4_^2^ = 531.08, *P* < 0.001, Figure 6(b)). Surprisingly, the POHD baited with CO_2_ + Be attracted and killed higher proportion of mosquitoes than POHD baited with fresh strips of Mb (*z* = 11.89, *P* < 0.001), nine-month-old strips of Mb (*z* = 6.96, *P* < 0.001), with Be (*z* = 14.11, *P* < 0.001), and without Be (*z* = −14.91, *P* < 0.001). However, POHD treated with fresh strips of Mb blend attracted and killed more mosquitoes than POHD baited with nine-month-old strips of Mb (*z* = 3.58, *P* < 0.01), with Be (*z* = 11.89, *P* < 0.001), and without Be (*z* = −12.85, *P* < 0.001, Figure 6(b)).

In the POHD baited with Ifakara blend in microencapsulated polymer pellets (granules), the proportion of attracted mosquitoes varied significantly with treatments (*χ*_5_^2^ = 631.49, *P* < 0.001, Figure 7(a)). The POHD baited with fresh Ifakara blend in granules attracted and killed significantly higher proportion of mosquitoes than the POHD baited with Ifakara blend six months (*z* = 6.41, *P* < 0.001) and nine months after impregnation in granules (*z* = 9.01, *P* < 0.001), CO_2_ + Be (*z* = 7.16, *P* < 0.001), with Be (*z* = 15.24, *P* < 0.001), and without Be (*z* = −15.50, *P* < 0.001, Figure 7(a)). The POHD baited with the blend six months after impregnation attracted and killed higher proportion of mosquitoes than the POHD baited with the blend nine months after impregnation (*z* = −3.07, *P*=0.02). POHDs baited with the blends either six or nine months after impregnation attracted and killed higher proportion of mosquitoes than the POHD with and without Be (*P* < 0.001). The proportion of mosquitoes attracted to and killed by POHD with CO_2_ + Be was similar to that of POHD baited with either 6 mo (*z* = −0.86, *P*=0.95) or 9 mo old blends (*z* = 2.23, *P*=0.22). There was no significant difference in the proportion of dead mosquitoes from the POHD with and without Be (*z* = −1.94, *P*=0.37, Figure 7(a)).

In the POHD with Ifakara blend in soaked nylon strips, the proportion of mosquitoes attracted to the POHD varied significantly with treatments (*χ*_4_^2^ = 579.07, *P* < 0.001, Figure 7(b)). The POHD baited with fresh strips of Ifakara blend attracted and killed significantly higher proportion of mosquitoes than POHD baited with nine-month-old strips (*z* = 6.45, *P* < 0.001), CO_2_ + Be (*z* = 3.38, *P* < 0.01), with Be (*z* = 14.27, *P* < 0.001), and without Be (*z* = −15.07, *P* < 0.001). However, POHD baited with nine-month-old strips also attracted and killed higher proportion of mosquitoes than the POHD with Be (*z* = −9.76, *P* < 0.001) and without Be (*z* = −11.16, *P* < 0.001, Figure 7(b)). The POHD with CO_2_ + Be killed more mosquitoes than POHD baited with nine-month-old strips (*z* = 3.26, *P* < 0.01), with Be (*z* = 12.16, *P* < 0.001), and without Be (*z* = −13.19, *P* < 0.001, Figure 7(b)). The POHD with Be killed significantly higher proportion of mosquitoes than POHD without Be (*z* = −2.86, *P*=0.03).

## 5. Discussion

The present study clearly demonstrates improvement in the efficacy of POHD with regard to placement of blends and mosquito entry point. The POHD with bottom placed blends and mosquito entry was relatively more attractive than the POHD with top placed blends and mosquito entry. The increased attractiveness in POHD with bottom placed attractive blends and mosquito entry could have been contributed to relatively higher release rate of CO_2_ and blend to the outside of the device. Similar observations were also reported in other studies although with slightly different setups and conditions. The Mosquito Magnet X trap (MMX) and Suna trap with bottom placed attractants and mosquito entry point attracted relatively high proportion of mosquitoes [16, 21, 48]. The POHD with top placed blends and mosquito entry created a long path of plumes by first flowing upward then downward, thus compromising the strength and release rate of odour plumes [49, 50]. Similar explanation was responsible for low catches in homemade trap [5].

The treatments in POHD with bottom mosquito entry indicated that a combination of Mbita blend and CO_2_ attracted significantly higher proportion of mosquitoes than Mbita blend alone. This finding corroborates with many previous studies which showed that traps baited with combination of CO_2_ and synthetic human body odour caught proportionally large number of mosquitoes [26, 29, 30]. This emphasizes that bottom placement of synthetic blends and CO_2_ improves attractiveness of the POHD to biting mosquitoes. With such placement, the natural air flow would sufficiently disseminate attractants outside the device and attract a considerable proportion of mosquitoes.

On the other hand, the attractiveness of improved POHD was strongly dependent on the type of blends, substrate/vehicle (granules and nylon strips), and residual activity of the incorporated blends. Mbita blend attracted significantly higher proportion of mosquitoes than Ifakara blend irrespective of the type of substrate used. The greater attractiveness of Mbita blend may be hypothesised to be attributed to the possibility that the volatile compounds in Mbita blend disperse more readily than those in Ifakara blend. This finding in our study agree with that of a previous study indicating that Mbita blend attracted relatively higher proportion of *Anopheles gambiae* s.l. and *An. funestus* than Ifakara blend [37]. Furthermore, the POHD baited with Mbita or Ifakara blends impregnated in granules attracted greater proportion of mosquitoes than that baited with either blends impregnated in nylon strips. The influence of substrate on the attractiveness of mosquito odour blends has repeatedly been demonstrated in other studies [29, 37, 38, 47, 51]. For example, traps baited with nylon strips of Ifakara blend were more attractive than those baited with its liquid formulation in glass vials or low density polyethylene (LDPE) [29, 38]. Similarly, traps baited with attractants impregnated on cotton, polyester, and cellulose polyacrylate materials were more attractive than those with attractants in soaked nylon strips [48]. The observed variation in the current study between nylon strips and granules could be explained by the fact that the porous materials in the granules provide more effective adsorbing capacity which subsequently allows equal and efficient deliberation of the odours to the environment. Granules have delivered entomopathogenic bacteria [52] and fungi [53–56].

Moreover, the attractiveness of improved POHD was influenced by the residual activity/persistence of applied blends. Fresh Mbita and Ifakara blends attracted significantly greater proportion of mosquitoes than the older ones (six and nine months after preparation) irrespective of the type of the substrate used to deliver them. Fresh odour blends of different compounds attracted significantly greater number of mosquitoes than the older ones [57]. Although the nine months' blend attracted significantly fewer mosquitoes, the proportion was yet acceptably high and comparable to the findings of several other studies [58, 59]. Synthetic blends consistently attract mosquitoes for up to 1 year after treatment under semifield conditions [58, 59]. Similarly, BG lures applied on granules remained attractive to *Aedes* mosquitoes for up to 5 months after treatment [27]. The residual attractiveness of blends declines over time due to the activity of bacteria [58, 59]. Results of the current study suggest that Mbita blend in microencapsulated pellets/granules may retain attractiveness to mosquitoes even beyond nine months of repeated use under semifield conditions.

The improved POHD has implications on both the control of residual malaria transmission and management of insecticide resistance in mosquito vectors. Being passive and portable and permitting combination of insecticides through mosaicking/rotation, the POHD could serve as a resistance breaking tool [60, 61]. The POHD offers a promising platform for applying novel insecticides such as carbamates (bendiocarb), pyrroles (chlorfenapyr), and other compounds that are not recommended for use on bed nets. Furthermore, the POHD will allow application of chemical insecticides in powder formulation via unique electrostatically charged netting. Insecticides applied in powder form have proven effectiveness against mosquitoes that are resistant to wettable formulations of the same insecticides [46, 62]. Moreover, the POHD will permit the combination of such insecticides with biological control agents like entomopathogenic fungi, bacteria, and viruses. The combination of insecticides with unrelated modes of action has demonstrated huge value in reducing mosquito population and disease transmission risk in many disease endemic countries particularly in Africa [63, 64]. The combination of chlorfenapyr sprayed walls and treated netting was reported to kill high proportion of outdoor biting [65] and insecticide resistant malaria vectors [4].

Despite the promising findings, the improved POHD has a number of limitations that need to be addressed to enhance its efficiency. The study was conducted under the semifield conditions using population of *An. arabiensis*. Although this vector species dominates the transmission in sub-Saharan Africa [66–69] efficiency of the POHD against the wild population of *An. arabiensis* may differ from what was observed in the current study. The synthetic blends were stored under refrigerator temperature (−4°C) between experiments. Such artificial climatic conditions are certainly far different from the reality. Therefore, evaluation of the blends under natural field conditions is desirable. Like other odour-baited devices, the POHD will depend on synthetic CO_2_ from cylinders or buckets containing a recipe of warm water, molasses, and yeast [18, 29, 48, 58]; therefore, further research geared at devising novel alternatives of CO_2_ source is inevitable. Further studies are required to assess the effects of ratio of yeast and molasses in the mixture on the types and quantity of volatile organic compounds and their role in enhancing attractiveness of CO_2_-baited POHD to mosquitoes. With the existing scare source of CO_2_ for large-scale surveillance and control of mosquitoes, our subsequent studies assessed the potential of using alternative compounds that mimic CO_2_ in attracting mosquitoes. Lastly, the POHD has poor trapping mechanism; thus, during the study, some of the mosquitoes could have entered and left the device without contacting the insecticide (used as the proxy to determine proportion of mosquitoes that visited the device). Therefore, there is a need to improve trapping mechanism of the POHD. This would render a dual purpose POHD, sampling and control of outdoor biting mosquitoes.

In conclusion, the findings of this study imply that the attractiveness of improved POHD was influenced by placement of attractants and bioactives. The residual activity of synthetic blends varied with the type of substrate/vehicle into which they were carried for delivery to mosquitoes. The shelf life of blends in microencapsulated pellets (granules) was longer than that of blends in soaked nylon strips. These findings warrant further evaluation of the POHD under real life conditions.

## Data Availability

The data used to support the findings of this study are available from the corresponding author upon request.
